# Interleukin-35 on B cell and T cell induction and regulation

**DOI:** 10.1186/s12950-017-0164-5

**Published:** 2017-08-07

**Authors:** Ai Huang, Lin Cheng, Miao He, Jun Nie, Jianjun Wang, Ke Jiang

**Affiliations:** 1 Department of Thoracic Surgery, Union hospital, Tongji Medical College, Huazhong University of Science and Technology, No.1277 Jiefang Avenue, Wuhan, Hubei Province 430022 People’s Republic of China; 2 Department of Anesthesiology, Union hospital, Tongji Medical College, Huazhong University of Science and Technology, No.1277 Jiefang Avenue, Wuhan, Hubei Province 430022 People’s Republic of China

**Keywords:** Interleukin −35, B cells, T cells

## Abstract

Interleukin (IL)-35 is a relatively newly discovered member of IL-12 cytokine family that is unique in that it is a dimer formed by two subunits. The review documents the structure, secretion and signal transduction of IL-35, the regulation effect of IL-35 on B cells and T cells as well as the adoptive transfer of IL-35+ regulatory B cells (Breg), therapeutic prospects of recombinant IL-35 (rIL-35) and IL-35 regulation role in various diseases. B-cell regulation expands the regulatory range of IL-35 and alters the view that IL-10 is the chief immune mechanism for Breg cells which secrete IL-35. IL-35 induces Breg cells, which then can induce Treg cells. IL-35 also plays an immunomodulatory role in the human body.

## Background

Interleukin (IL)-35 cytokine is a relatively newly discovered member of IL-12 family which are unique in structure as they are dimer formed by two subunits. Existing family members (IL-12, −23, −27) are similar in structure, receptor binding, and downstream signaling pathways that positively or negatively regulate the immune system. IL-35 also strongly inhibits immune function and this review will state the structure and secretion of IL-35, its effect and regulation in B cells and T cells as well as the therapeutic prospects of recombinant IL-35 (rIL-35) and IL-35 regulation role in various diseases.

### Interleukin-35 (IL-35) structure and secretion

IL-35 was found by Niedbala [1] and Collison [2] almost simultaneously and it is reported to contain IL-12α chain p35 and IL-27β chain Epstein-Barr virus-induced gene 3 (Ebi3) connected by disulfide bond. IL-35, initially named at the 13th International Congress of Immunology, is the new focus of cytokines research. IL-35 is similar to other IL-12 family members which are heterodimeric glycoproteins formed with disulfide-linked α (p19, p28, or p35) and β (p40 or EBi3) chains. The α-chain has 4-α-helical bundles, a typical cytokine structure, and the β-chain is homologous to the soluble cytokine receptor. p35 and p40 combine to form IL-12; p19 and p40 combine to form IL-23; p28 and Ebi3 combine to form IL-27 [3]. IL-35 is composed of p35 and Ebi3, and it differs from the expression and secretion way of other IL-12 members. In response to bacteria, bacterial products, or intracellular parasites, IL-12, IL-23, and IL-27 are secreted by activated antigen-presenting cells, including B cells, monocyte, macrophages and dendritic cells [4–6]. IL-35 was initially reported to be produced by Treg cells and was essential for maximizing the inhibitory role of Treg cells [2]. Recently studies suggest that regulatory B cells (Breg) also produce IL-35 and rIL-35 fusion proteins can induce Breg cells to secret IL-10 and IL-35 [7, 8].

### IL-35 receptors and signal transduction

**Fig. 1:**
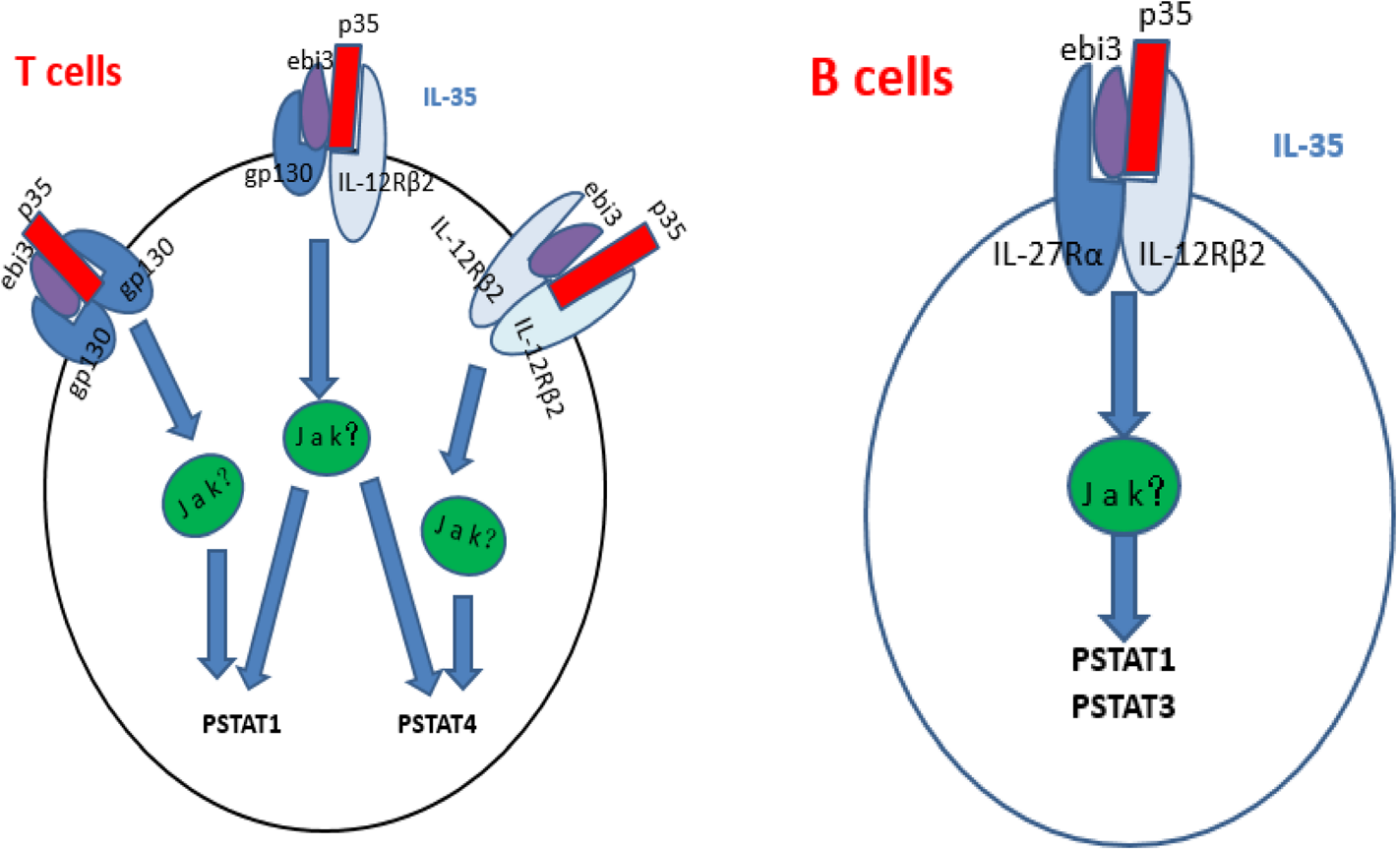
IL-35 signal transduction in T and B cells. Signaling through gp130 or IL12Rb2 homodimers to STAT1 or STAT4 separately in T cells, while formation of the gp130/IL12Rb2 heterodimer is required for both STAT1 and STAT4 activation in Treg cells (*left panel*). B cells respond to IL-35 through the IL27Ra/IL12Rb2 heterodimer to STAT1 and STAT3 (*right panel*)

Peptide chain sharing is common to the IL-12 family as they bind to receptors to activate signal transducer and activator of transcription (STAT) proteins [9]. IL-35 is uniquely anti-inflammatory cytokine in contrast to other IL-12 pro-inflammatory cytokines [9]. This difference is thought to be associated with the receptors and signaling pathways specific to IL-35 and future studies should confirm these assertions. An obstacle to understanding the molecular mechanism underlying IL-35 is the lack of clarity about the IL-35 receptor (IL-35R) and its signal transduction pathway [9]. Collison’s group reported that mouse IL-35R differed from traditional cytokine receptors. IL-35R is composed of dimers which are IL-12Rβ2 homodimers, gp130 homodimers or IL- IL-12Rβ2/gp130 heterodimer [6]. IL-35 binds to IL-35R and initiates signal transduction and exert biological function. IL-12Rβ2 or gpl30 homodimer activates STAT4 or STAT1, but only IL-35R in the IL-12Rβ2/gp130 heterodimer form can induce STAT1- and STAT4-activated signaling pathways to mediate Treg cell function and positively or negatively feedback regulate IL-35 gene expression [2]. IL-35 is reported to activate STAT1 and STAT4 in T cells [7] where IL-35 binds to gp130 and IL-12Rβ2 receptors [2], both of which depend on JAK-STAT signaling to introduce intracellular responses. However in B cells, IL-35 signaling mediates STAT1 and STAT3 activation through IL-12Rβ2: IL-27Ra heterodimers [8] (Fig. 1).

### IL-35 regulation and signal transduction in Breg cells

B cells have been traditionally thought to contribute to immune defense by secreting antibodies and antigen presentation but they also have function in immune regulation as Breg cells [10]. There are multiple B cell subsets which have immune regulation function, such as CD138+ plasma cells [11], B10 cells (CD1d^hi^CD5^hi^) [10], CD21^hi^CD23^hi^CD24^hi^ transitional 2-marginal zone precursor cells [12], and Tim-1^+^ B cells [13], but Breg cells do not have a unified determined phenotype. It is generally recognized that Breg cells play a role in immunosuppressive function by secreting IL-10 under the stimulation of toll-like receptor (TLR) agonists, CD40L, and IL-21 [14].

**Fig. 2:**
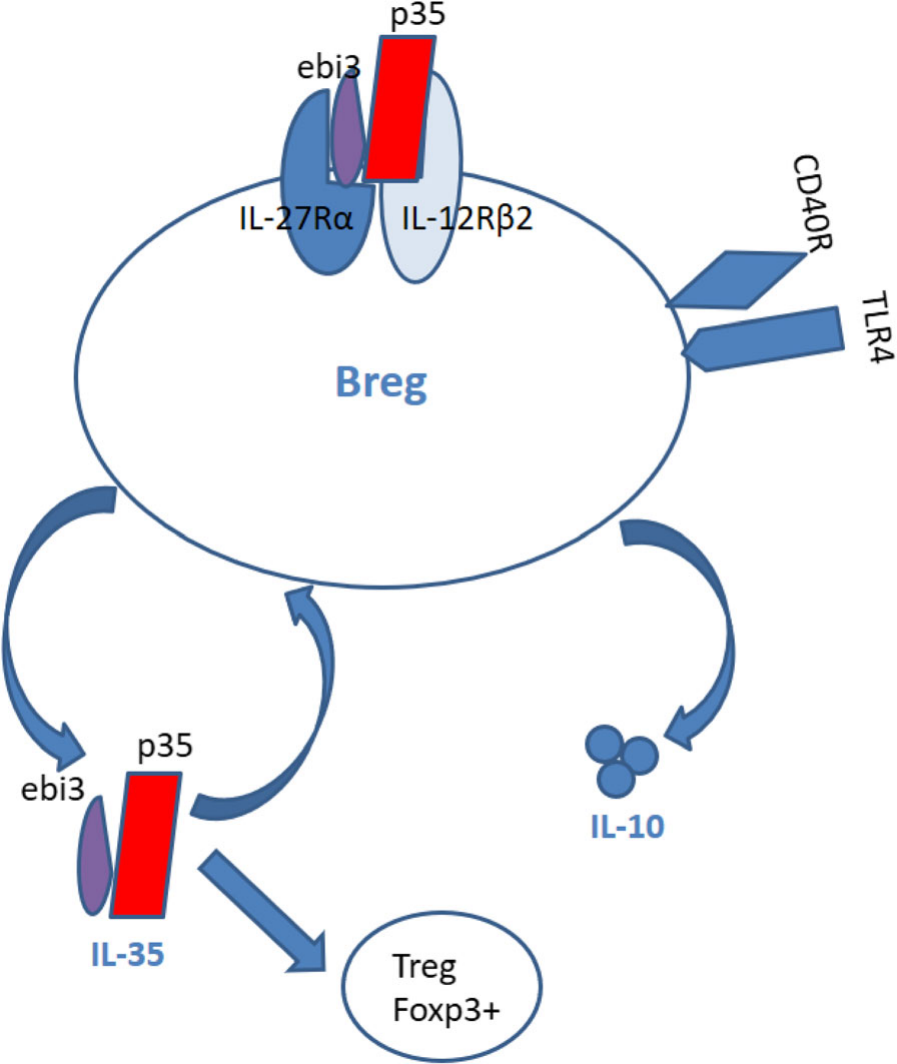
IL-35 regulation in Breg cells. The immunosuppressive cytokine IL-35 induces the expansion of Breg cells, which secrete IL-35 to protect from autoimmune disease. Secretion of B cell–derived IL-35 seems to have an autocrine role through activation of the IL-35 receptor (IL-12Rb2 and IL-27Rb) to expand or induce Breg cells. B cell–derived IL-35 also acts on T cells to induce a Foxp3+ Treg cell population

Furthermore immunosuppressive pathways of Breg cells may not solely depend on IL-10 [15] as research suggests that Breg cells can produce IL-35 and that rIL-35 can induce Breg cells to secrete IL-10 and IL-35 [7, 8]. B-cell-derived IL-35 also acts on T cell-induced Foxp3^+^ Treg cells [7, 15] (Fig. 2). Although rIL-35 inhibits B220^hi^ B cell proliferation, it selectively induces CD19^+^CD5^+^B220^lo^ Breg cell proliferation in vivo [7].

Unlike T cells, inhibition of gp130 (with small interfering RNA [siRNA] or neutralizing antibodies) does not affect IL-35-mediated of B cell proliferation or IL-10 secretion. In contrast, silence of IL-12Rß2 and IL-27Ra in B cells completely blocks inhibition function of IL-35. Thus IL-35 may mediate biological activity in different cell types via different receptors and STAT signaling pathways and future studies are required to confirm those [14].

### IL-35 regulation and signal transduction in T cells

IL-35 is a novel inhibitory cytokine that may be specifically produced by Treg cells. It is required for maximal suppressive activity of Treg. In addition, IL-35 can block the proliferation of Th1 and Th17 cells by limiting early T cell rest on the G1 phase of cell division [16]. Although IL-35 can inhibit Th1 proliferation, it is resistant to Treg conversion due to the potent inhibition of Ebi3 and p35 transcription by IFNγ from Th1. Moreover, IL-35 blocks Th2 development by repressing GATA3 and IL-4 expression and limiting Th2 proliferation. IL-35 can also mediate conversion of Th2 cells to Treg, although this can be blocked by IFN-γ [17].

**Fig. 3:**
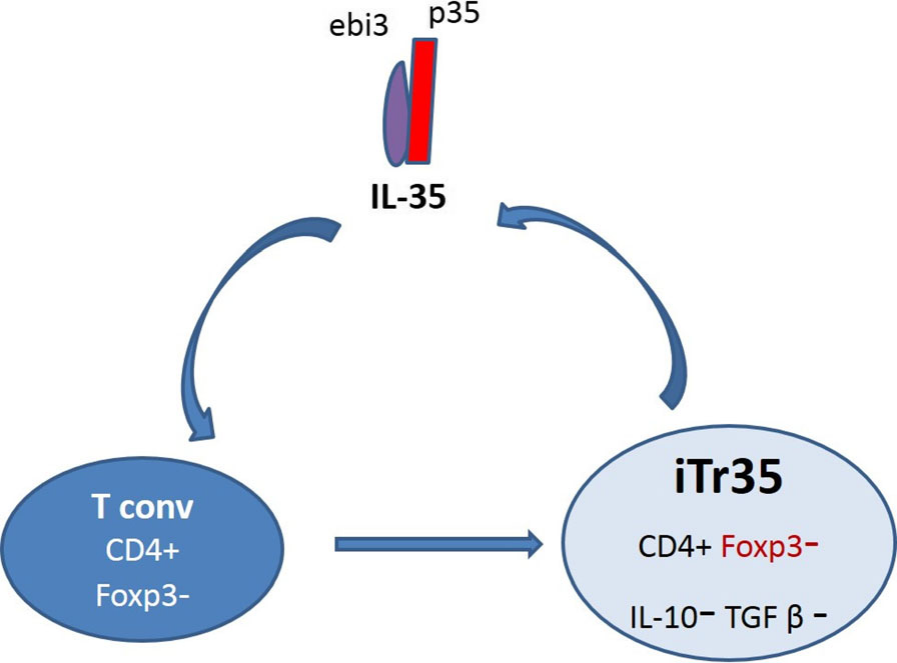
iTreg generate. Naïve human or murine T cells were stimulated with IL-35 and converted into a regulatory population – iTr35 cells – which exhibit a highly restricted gene signature (CD4 + Foxp3 − EBI3 + p35 + IL10 - TGFβ −).

Like TGF-β and IL-10, IL-35 can induce the development of an induced regulatory T cell (iTreg) population, iTr35, which suppressed T-cell proliferation via IL-35 [2]. iTreg do not express Foxp3, IL-10, and TGF-β (Fig. 3). iTr35 were as effective as nTregs at restoring immune homeostasis and preventing autoimmunity disease in Foxp3^−/−^ mice by limiting the proliferation of T cells and so prevented the modeling of EAE and IBD and promoted the proliferation of B16 tumor cells. Adoptive transfer IL-35 treatment increased the proliferation of Foxp3^+^CD39^+^ CD4^+^ T cells which secreted IL-10 for autoimmune protection in a collagen-induced arthritis model [18]. However, whether the presence of IL-35 can mediate Treg amplification under physiological conditions is still uncertain.

While gp130 is fairly ubiquitously expressed, IL-12Rβ2 is expressed mainly on the surfaces of activated T cells, natural killer cells, B cells, and dendritic cells [19]. IL-12Rβ2 is undetectable on most resting T cells, but can be rapidly up-regulated by exposure to IL-2, IFN-γ, IL-12, IL-27, and TNF-α. Indeed, IL-2 or IL-27 pretreatment increases T cell sensitivity to IL-35 mediated suppression [9].

IL-35 mediates the inhibitory effect on T cells through the signal pathway of STAT1 and STAT4, but also lead to the pro-inflammatory effects by activating the STAT molecule of IFN-γ and IL-12, in which the key difference is that IL-35 induced STAT1-STAT4 heterodimer formation [20, 21].

### Recombinant IL-35

Highly purified heterodimeric cytokine IL-35 is difficult to obtain and this is a limitation to immunology research, especially for elucidating the role of this cytokine in autoimmune, infectious diseases and tumor immunity. Transgenic technology has been applied to infect cells to establish a rIL-35 mouse model in which rIL-35 is a heterodimer of p35 and Ebi3 [7]. Similar to IL-27, IL-35 is not secreted as a disulfide-linked heterodimer as Ebi3 associates non-covalently with the IL-12p35 [22]. The native Ebi3/p35 heterodimer would be difficultly to isolate in vivo because only about 4% of the secreted Ebi3 co-precipitated with the IL-12p35 in vitro over expression studies in COS7 cells [22]. Meanwhile, as the absence of IL-12p35 substantial amount of the Ebi3 degraded in the ER (endoplasmic reticulum), thereby reducing the bioavailability of ebi3. These contribute to the low levels of IL-35 in vivo [23]. Others generated recombinant mouse IL-35 (rIL-35) using a bicistronic vector containing IRES (internal ribosomal entry site) that allowed stoichio etric expression of the Ebi3 and IL-12p35 [2]. Another approach that has been used is to construct a heterodimeric protein covalently linking Ebi3 and IL-12p35 [24].

### IL-35regulation in various diseases

(1) IL-35 and autoimmune diseases.

Early studies mainly used a model of p35 or ebi3 deficient mice. However, the absence of IL-12α affects the function of IL-12 as well as that of IL-27 and IL-35. Despite these complexities, Ebi3−/− and Il-12a−/− mice model also provide the proof that IL-35 have immune suppressive function in some autoimmune diseases such as experimental autoimmune uveitis (EAU) [7], experimental allergic encephalomyelitis (EAE) [2], collagen-induced arthritis (CIA) [25], encephalitis [26], and other conditions. That IL-35 fusion protein obtained from genetic engineering produced data consistent with the knockout model results and studies indicates that injection of rIL-35 can be used to protect against allergic airway model [27] as well as colitis [20] and autoimmune diabetes [28]. Studies show that IL-35 regulates immune function via T cells in many diseases. In NOD transgenic mice, infusion of IL-35 over expressing islet cells significantly alleviated symptoms of diabetes and local inflammatory responses, reduced the number of effector CD4^+^ and CD8^+^ T cells in pancreatic islet tissue, inhibited infiltration of T cells from transitioning from G1 to S phases, and decreased effector T cells proliferation [28]. In a type II CIA model, infusion of rIL-35 reduced local inflammatory responses by inhibiting Th17 cell differentiation. In an Ebi3 subunit knockout mouse model, enhanced Th17 cell infiltration and aggravated inflammatory response was noted [1].

**Fig. 4:**
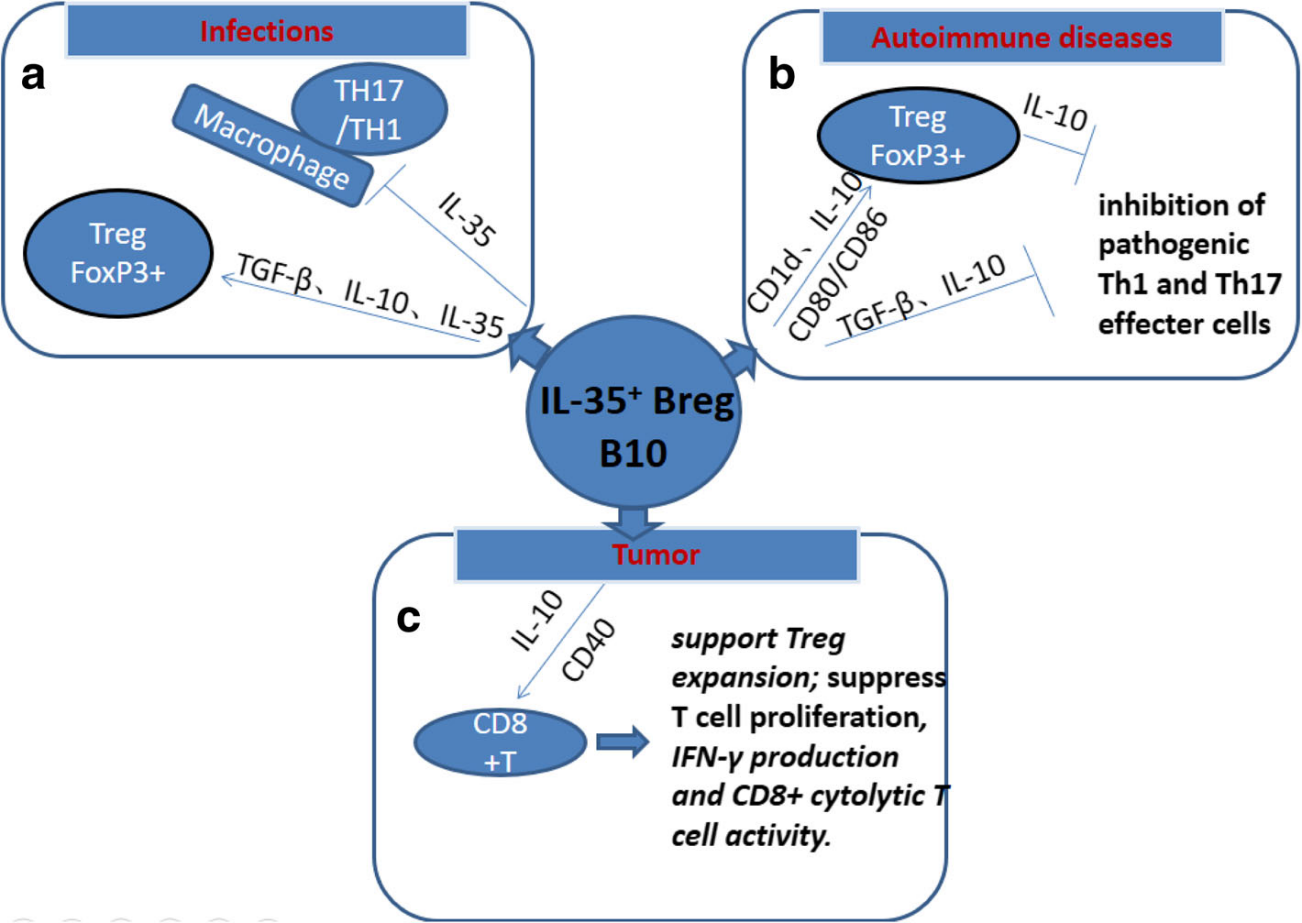
**a** B cell–derived IL-35 can suppress T-cell and monocytic responses. IL-10, IL-35, and TGF-β induce the Treg cell population. **b** Breg cells activate Treg cells through IL-10 production and B7 costimulatory molecules. Activated Treg cells release IL-10, suppresses the autoreactive Th1response in patients with EAE. IL-10– and TGF-β–producing Breg cells can suppress T-cell responses. **c** A subset of Breg cells also acquires the ability to produce IL-10. Breg cells from the tumor bed support Treg expansion in vitro and in vivo, suppress T cell proliferation in vitro, suppress IFN-γ production and inhibit CD8+ cytolytic T cell activity. These B regulatory properties inhibit the anti-tumor response and lead to enhanced tumor growth

To study a potential regulatory role of IL-35+ Breg, a model of EAU was established and rIL-35 or IL-35+ Breg could control the incidence and development of EAU. IL-35+ Breg cell-mediated protection depended on induction and proliferation of endogenous Breg cells and Foxp3+ Treg cells and inhibition of pathogenic Th1 and Th17 effector cells [7]. Adoptive infusion of rIL-35-mediated Breg cells improved the animal health after EAU eradication as well. This suggested that in vitro study of functional rIL-35+ Breg cells may give insight into potential roles for Breg and IL-35 Breg cells in autoimmune disease and cancer. IL-35-derived Treg cells (i.e., iTR35) can maintain Breg cells, but IL-35+ Breg cells can also induce Treg cells. Therefore, in an inflammation-derived IL-35-dependent regulatory environment, different cell populations generated by IL-35 may interact via positive feed forward mechanisms and induce iTR35 and IL-35 + Breg cells through infectious tolerance [7] (Fig. 4).

#### IL-35 and tumor immunity

Previous work suggests that IL-35 plays a role in tumor immune escape. Ebi3 expression was increased in Hodgkin’s lymphoma [29] and more Treg cells and inhibitory cytokines were noted in peripheral blood and tumor microenvironments of patients with pancreatic or breast carcinomas [30]. Ebi3 expression in lung cancer cells has also been found to be associated with tumor progression and siRNA-mediated down-regulation of the Eib3 gene inhibited proliferation of lung cancer cells [31]. Similarly, inoculation of b16 tumor cells in Ebi3-knockout mice showed enhanced anti-tumor immunity relative to wild-type mice and the metastatic potential of tumor cells were suppressed. In an IL-35-positive tumor cell microenvironment, studies showed that there were significant increase in CD11b + Gr1+ myeloid-derived suppressor cells (MDSC) and vascular endothelial growth factors promoted tumor angiogenesis [32]. These MDSCs were immunosuppressive and inhibited cytotoxic T cells. In this way, tumor cell-generated IL-35 may protect against cytotoxic T-cell-mediated damage. In contrast, applications of neutralizing antibody to IL-35 significantly inhibited tumor cell proliferation [32].

Although many studies have shown that IL-35 contributes to immunosuppressive function in the mouse model, but this effect is limited in humans [33]. No expression of IL-35-derived Treg cells had been observed in early investigations in humans. This suggests that IL-35 may not be constitutively expressed in humans. However, it can be produced in specific tissues and cell types after a certain inflammatory stimulus. Recent studies have shown that, under strong stimulation, Treg can produce IL-35, and CD4^+^ T cells under the stimulation of IL-35 can express IL-35similar to iTr35 in mice [34]. Similarly, in humans, CD8^+^ Treg can suppress the immune response against prostate cancer by expressing CTLA-4 and IL-35 [35]. Although more research is required to draw any conclusions, preliminary information suggests that elevated serum IL-35 is associated with tumor malignancy [36–38] and clinical stage and decreased IL-35 is associated with autoimmune disease and chronic infection [39–41]. (Fig. 4).

#### IL-35 and infectious diseases

IL-35, as an inhibitory cytokine, plays an important role in infectious diseases. The study suggested that Mycobacterium tuberculosis (M. tuberculosis) could induce T cell proliferation and foster IFN-γ production in p35-deficient mice, P40 deficient mice, and wild-type mice, thus eliminating the pathogen. Mice lacking p40 have been found to be less able to show antigen-specific responses than those lacking the p35 subunit. However, their ability to counteract infection is less pronounced than that of wild-type mice [42]. In addition, the protective responses can be induced in wild-type and p35-deficient mice by inoculating with vaccines, and increased the secretion of IFN-γ and IL-17. However, P40-deficient mice did not produce antigen-specific IFN-γ or IL-17, and increased the infection load of bacteria. In addition, treatment of p35-deficient mice with *Candida albicans* (*C. albicans*) reduced the rate of fungal infection and was associated with little to no obvious symptoms of infection relative to P19-deficient mice. In p35-deficient mice and those in which p35 gene was disrupted, the immunosuppressive function of IL-35 was inhibited, which impaired anti-fungal immunity [43]. The CD4^+^ T cells in the peripheral blood of patients with chronic hepatitis B also showed high levels of expression of p35 and EB13 protein, indicating that IL-35 was related to the immune response of chronic hepatitis B patients [44]. These results showed that, during the acute infection process, IL-35 preferentially activated Th1 cells, stimulated proliferation of Treg cells, and inhibited the differentiation of Th17 cells, thereby preventing excessive tissue damage caused by the clearing of pathogens. In the chronic infection and inflammation, IL-35 selectively inhibited effector cells, including Th17 cells, which slowed down the development of autoimmune diseases [1]. (Fig. 4).

## Conclusions

B-cell regulation expands the regulatory range of IL-35 and alters the view that IL-10 is the chief immune mechanism for Breg cells which secrete IL-35. IL-35 signaling mediates STAT1 and STAT3 activation in B cells by binding to IL-12Rβ2 and IL-27Ra receptors and inducing Treg cells which provide mutual stimulation under inflammation and amplification of regulatory cells. IL-35-induced B cells also transform to secretary IL-35^+^ Breg cells and rIL-35 or IL-35^+^ Breg cells control the incidence and development of EAU. IL-35 induces Breg cells, which then can induce Treg cells. In the case of inflammation, cells which can generate IL-35 provide mutual stimulation, resulting in amplification of regulatory cells. Recent researchers have found that in some inflammatory stimulus conditions, IL-35 also play an immunomodulatory role in the human body, so we can look forward to further exploring immunotherapy approaches through IL-35.

Future studies may address whether IL-35 shares its receptors with other IL-12 members and rIL-35 may help clarify these biological effects and identify other cell types or subtypes involved in immune regulation by producing IL-35. How IL-35 inhibits cell proliferation, why different cells mediate different signaling pathways, and potential prospects of clinical use of IL-35 (rIL-35 and IL-35 Breg cells) as a chemotherapeutic or to treat autoimmune diseases or organ transplantation also await further study.

## Abbreviations

Breg: Regulatory B cells
CIA: Collagen-induced arthritis
EAU: Experimental autoimmune uveitis
Ebi3: Epstein-Barr virus-induced gene 3
ER: endoplasmic reticulum
IL-35: Interleukin-35
IL-35R: IL-35 receptor
iTR35: IL-35-derived Treg cells
MDSC: Myeloid-derived suppressor cells
rIL-35: recombinant IL-35
siRNA: small interfering RNA
STAT: Signal transducer and activator of transcription
TLR: Toll-like receptor
Treg: Regulatory T cells
